# Graves’ Disease

**Published:** 1886-08

**Authors:** William C. Bailey

**Affiliations:** Albion, N. Y.; Member of Orleans Co. Medical Society; Central New York Medical Society; New York State Medical Association; American Medical Association, etc.


### *GRAVES' DISEASE.**

By WILLIAM C. BAILEY, M. D., Albion, N. Y.

Member of Orleans Co. Medical Society; Central New York Medical Society; New York State Medical Association; American Medical Association, etc.

In a lecture delivered before his pupils a few years ago, Dr. Jacobi observed, that so rare is exophthalmic goitre, and so comparatively few have been the cases reported especially among youth and children, the history of every one should be presented to the profession. Then, as regards disease not witnessed in every-day practice, it is well, occasionally, to refresh ourselves with the symptoms and the various forms in which they may be met. Too rarely has consideration

*Read before Central N. Y. Medical Society, Rochester, May 18, 1886.

of this subject been urged before medical societies. It is liable to occur in practice at any time, in a variety of ways, and due to a variety of causes. In its incipient stages, especially, a mistaken diagnosis and too grave a prognosis may readily be made. One is apt to become more interested in a problem of mathematics undemonstrated than in one which, to him, is plain and simple. Having solved one, he calls for another. Thus it is in studying disease. The physician attends such gatherings as this, not only to learn of new things regarding cases he commonly meets, but also, that he may at least receive an impulse to study more fully something in his art not altogether understood—some problem not so near solution as others.

The history of a case illustrating the manner in which exophthalmic goitre may present itself, is as follows:

Miss A—— is the sixth in a family of children of that number, whose parents were of strong, sturdy, English and Irish extraction. No hereditary taint can be found; but an older sister had bronchocele, which developed when she was fourteen, and disappeared after about four years. At the age of thirteen, Miss A—— applied to a local physician for advice in reference to a swelling of the right thyroid. The doctor remarked to her, during examination, that she had the fastest pulse he had ever known in one able to be about. He advised hospital treatment, and predicted serious results. In two months, she called again. He found the same condition, and gave the same advice. No medicine was prescribed or taken. Feeling well, and supposing the enlargement to be simple goitre, which would disappear as in her sister's case, she attended school, studied hard, developed well, with organs normal, and weighing over 130 pounds. Her mother recalls, however, even before the appearance of the goitre, that running, or any violent exercise, would produce palpitation noticeable at a distance. Such was her condition, when, three years later, she contracted a severe cold, and Feb. 6, 1885, sent for medical aid. The writer found her very ill with marked lung congestion, and coughing almost incessantly. T. 103°, R. 28, and P. entirely imperceptible in the wrist. By aid of the stethoscope, a valuable means of obtaining an accurate count in great rapidity, the pulsations were found to be 168. This, with the other symptoms and extreme weakness, alarmed me, and my anxiety was communicated to the family. Treatment was directed toward relieving lungs and cough, giving nourishment and plenty of stimulants. Next morning found her presenting the same general characteristics, save that the cough was less, and the T. had fallen one degree. R. 28, P. 168, and still no throbbing in the wrist. The extremities cold and clammy yesterday, were colder and bluer to-day, and to prolong life seemed scarcely possible. The stimulants were increased, warmth thoroughly applied, and toward night she was seen again. The following day there was slight improvement observable, and this now gradually progressed, so that, Feb. 17th, the temperature was 99½°, the lung congestion had yielded, the appetite had returned, and much strength had been gained; but the pulse remained the same, ranging from 164 to 190, was weak, and no trace could be detected in the radial. The extremities were cold and apparently bloodless until the 23d. Now, for the first, the temperature was normal, and so remained. All symptoms were better; the pulse 168, and felt slightly in the wrist. Stimulants were lessened as she improved. Their value in this case could not be over-estimated; they were essential and highly beneficial. There was no gastric difficulty, and food was readily assimilated. No nervous disturbance was apparent at this time, nor afterward—a very unusual exception in this affection. Although the heart beat at the enormous rate indicated, she complained of no choking, no palpitation, no indication whatever of cardiac disorder; nor could there be heard, at any time, abnormal sounds. The congestive disturbance having lessened, with no reduction in pulse, the writer looked elsewhere some days before to account for it. Her history, the enlarged thyroid, the absence of organic indications in the heart, *the fact that she could recover*

*from a serious illness with such a heart-pulsation*, compelled me to recognize a clear case of Graves' disease, its inception dating back four years nearly, and now aggravated by the present lung-affection. Yet there were noticeably absent many diagnostic signs. Measurement of the neck was, at this time, sixteen and one-half inches; much less, she said, than formerly.

Feb. 27th, she first sat up, but soon fainted. Pulse was 94, irregular, strong and intermittent. The stethoscope revealed only the peculiar diagnostic thrill in the thyroid, which Dr. Hammond likens to the sensation felt when stroking a purring cat. Vomiting was severe, the extremities were cold, the face was deathly white, dyspnœa was marked, and there was great pain in the epigastric and cardiac regions. As she rallied shortly by the aid of stimulants, the pulse increased in rapidity, and was soon again 166. These attacks curiously recurred periodically every one or two weeks for about five months, and then disappeared.

As she grew stronger and could sit up, the heart's action gradually increased, and, March 13th, now readily found in the wrist, was as follows: Lying, 157; sitting, 166; standing, 192; walking to window, 212. With this marked difference, I advised rest in the recumbent position, but she was as full of fun and mischief as a boy, and would not remain quiet. The appetite, too, was excessive, a not unusual accompaniment in this disease. Following was the amount taken March 12th: Six pickles, ten slices of bread with butter, two biscuits, two teacupfuls of mashed potatoes, and one of pot-pie, one piece of meat, four inches square, one ounce of maple syrup, one apple, two quarts of tea and milk, one pint of water, six ounces of whiskey, and two of olive oil. This was a daily average, which I unsuccessfully attempted to correct.

There was not observable, at any time, the slightest exophthalmia, but considerable dilatation of the pupils was present for some months. The pulse remaining unchanged under the action of iron, aconite and largely increased doses of digitalis, the two latter were dropped, and veratrum was begun

April 3d. By May 20th, this had reduced the heart-beat to 105, but, on account of nausea and distress which it produced in necessarily increased doses, it had to be suspended. The pulse in a week was 192, once over 200. The veratrum, again tried, had no appreciable influence, possibly because only the smallest doses could be given without disturbance. It was then decided—Dr. Kittinger, of Lockport, and Dr. Squier, of Albion, examining the case about this time—to employ electricity, and following were the observations made the first time it was used: Continue iron in form of dialyzed. Came to office, walking two miles. Before sitting, the pulse was 198; after sitting five minutes, 168; after sitting one-half hour, 145; after reclining on couch ten minutes, 138; measurement of neck, 14¾ inches; weight, 112 pounds; feels very well; sleeps ten hours in each twenty-four. The Farradic interrupted current was used five to twelve minutes at a sitting, once in two or three days, gradually increasing length of *séance* to twenty minutes, and the number of days to five or seven. The anode was placed under the angle of the right jaw, and the cathode above the seventh cervical vertebra, according to the very successful method of Dr. A. D. Rockwell, of New York. Finally simple, general Farradization was shown to be even better, the temporary reduction of the pulse not infrequently being twenty beats per minute. Twenty-eight treatments were all that were given, with these results, noted November 1, 1885: Neck measurement, 13¾ inches; slight glandular enlargement still remains; pulse, 110; weighs 137 lbs. May 15, 1886: Has taken no medicine or treatment since last November; pulse, 92; is well and doing work at home for seven.

This affection is not wanting in names. They have varied according to supposed causes, as chlorosis, anæmia, struma, etc. It is most commonly known as Graves' or Basedow's disease, and exophthalmic goitre. Soelberg Wells suggests aneurysmatic bronchocele, and Walshe, cardiothyroid-exophthalmos. The last seems most appropriate, and the writer would make a plea in favor of its adoption. Here the three cardinal symptoms are recognized, and in their order of prominence. In "exophthalmic goitre," the name is defective, as it does not include that which is the first to appear, and the one almost never wanting, namely, the increased action of the heart; and it makes most prominent the exophthalmos, which is usually the last to present itself, and the one most frequently absent.

Flajani, Parri and Stokes described cases having the main symptoms of this disease most prominent, but Graves, of England, in 1835, and Basedow, of Germany, about the same time, were the first to recognize an individual affection. It is found mostly among adult women, rarely under the age of fourteen, or after middle life; but advanced age, men, and children are not entirely exempt. Some have regarded it as simply a cachectic condition; others have given a mechanical explanation of the phenomena; but it is now generally considered to be a neurosis, due to sympathetic irritation, principally in its cervical portion. "As the cardiac plexus governs the action of the heart, and the cervical ganglia of the sympathetic the branches of the carotids, any impairment of these centers would naturally produce dilatation" of the respective parts which each may control. This has been proven to be true from experiments by Goltz and others. For example, "section of the cervical sympathetic will cause dilatation of the vessels of the head and neck, and some protrusion of the eye-balls;" and galvanization, or irritation of the cervical end of the sympathetic, Stokes claims, will produce hypertrophy of the heart. The dilatation of these vessels we naturally would expect from vaso-motor paralysis; but Goltz has proven "it may be also due to irritation of the fibres of the sympathetic, which, when stimulated, will dilate blood-vessels," and this, says Jacobi, "best explains the symptoms belonging to the heart, and also to the unstriped muscles in the orbit and eye-lids." Some consider that the three cardinal indications have separate centers to be affected. This theory receives some confirmation in their want of relation to each other. Any may be absent, and those present may vary in severity as much as it is possible to do so. Dr. Russell's deductions in this respect are of great interest. "Of twenty-three cases, goitre was absent in six; of these six, palpitation was extreme in four, and proptosis existed in a great degree in one, was absent in one and moderate in the others. In four of the twenty-three, proptosis was absent. In one of these four, goitre was absent; in three, it was of large size; in three of the four, palpitation was severe; in one, moderate. The heart symptoms were present in various degrees in all of the twenty-three cases. In one with the proptosis absent, there was lachrymation; in three others, dilatation of the pupils; in two of these, the heart symptoms were severe; in one, the goitre was large." Dilatation of arteries and veins has been found in all parts of the body. The thyroid shows great development of blood-vessels, "interlacing with each other so as to distend it as water distends a sponge." There may be, but not usually, infiltration. Thus it is seen to differ from true goitre. One writer says: "At the point of development into bronchocele, it ceases ordinarily in exophthalmic goitre." But it is not supposed to be necessary that this irritation of the sympathetic shall be due to real structural lesion, for a limited number of post-mortems shows it is not always present; therefore, it may be purely functional, and such must be the condition of those who recover; so, if we may judge from the few fatal cases recorded, a small minority only has organic lesion. This division may, then, place the disease under two classes, the one due to organic change in the sympathetic; the other, to functional irritation. But we should not forget that there *may be* structural lesion in nerve cell, or fibre, beyond the power of detection by the most powerful microscrope; and that "the proliferations of connective tissue, or the gray infiltration found in the ganglia, or the enlargement and thickening of the cervical sympathetic," as recorded by Jacobi, *may be* the result of the disease, and not the cause. Lesions are not found in these parts alone; the main branches leading to the inferior thyroid and vertebral arteries, the brain, spinal cord and medulla, have been involved.

Ushered in from almost all causes of a neurotic character, often succeeding or accompanying some disorder indicating debility, occasionally, however, occurring suddenly from shock or traumatic origin, Graves' disease is recognized by what Hammond calls "the great pathological trinity"—three distinct and prominent symptoms, which, in their usual order of appearance, are palpitation of the heart, enlargement of one or both thyroid glands, and protrusion of the eye-ball. All may be present, and one or even two may be absent. Russell describes a case of the latter. There may be years or a few hours between their appearance, and either may present itself first. It may run its course very shortly, as in Solbrig's case, where "ten days were sufficient for its inception, progress and complete disappearance;" or, as in another case, it may be more or less severe for over thirty years; or, it may disappear only to return. Again, this triad may be, one or all, scarcely noticeable or extremely severe. There may be every variety and phase in this respect. The heart may palpitate slightly, or at a wondrous rate, with murmurs and abnormal sounds, with hypertrophy or dilatation, and pain and distress. The goitre may be small or large (Praël records one as having weighed a pound); or, it may even vary from day to day, or pulsate like an aneurism, "with thrills and numerous whirring noises." "It may be painful or painless, soft or hard, compressible or not." The exophthalmos may produce simply a stare, or even lie upon the cheek and burst. But there are symptoms which usually accompany this trinity, some diagnostic, and an endless variety of others, according to each individuality. Nervous impairment in some form is usually present, from insanity and epilepsy and mania to the mildest hysteria. "The radial pulse may be several beats per minute less than that of the heart," on account of feeble and unequal contraction of the latter. "Peripheral dilatation of the blood-vessels may occur, with a vibrating pulse in the meta-tarsal or the palmar arch." The heavy beatings of the heart may shake the whole frame. The gland itself, generally, pulsates synchronous with the heart, and it may be filled with various sounds that are a source of annoyance in the sufferer's ear. Another indication is disassociation of the upper eyelid with the corresponding ball. When one looks down, the lid naturally falls in unison with the ball, but in Graves' affection the ball may move, and the lid remain, or move but partially. The exophthalmos may often, by gentle pressure, be returned to its normal position, but it will remain only so long as the pressure is continued. A more frequent characteristic, perhaps, than is generally recognized, is dilatation of the pupils, especially in the absence of the proptosis. Dyspnœa, fainting, choking, lateral or general profuse perspiration, œdematous extremities, vomiting, excessive appetite or aversion of food, diarrhœa, hemorrhage, and periods of excitement due to the heart's action, are symptoms common to this affection; yet one may be remarkably free from any or most all of these. We can but wonder at the amount of apparent disturbance to the system in a case, for example, like that related, with a heart-rapidity of more than twice its normal work, and still remain so free from its influence. In fact, we may regard such as pathognomonic.

This disease is frequent in families having ordinary bronchocele. One sister of my patient had it. Of another, related by Greenamyer, the mother died of exophthalmic goitre, and five sisters had bronchocele, the youngest being born with it. Such cases are not few, and have led some to regard the one as being occasionally resolved into or supplanted by the other. I must confess, it is difficult to discard all belief that there exists an entire want of relationship between those two affections. In no less than ten recorded cases, I find both diseases co-existing in the same family. An interesting article might be written upon this subject.

Dr. Ely, of Rochester, having recently inquired whether it might be merely accidental, that the most severe cases he or his father had seen during the last twenty years were from this, Orleans county, the writer has taken occasion to communicate with those physicians longest in practice here, in regard to their experience. They recall eleven or twelve cases, but there have doubtless been more. One dates back nearly twenty years. Most of the others have been quite recent. Several physicians have never treated any. Of the number referred to, all have been quite marked; most have been severe, and at least three have died, one within a few months. One proved nearly fatal. The goitre was large, the pulse averaged 140, and the proptosis was so severe as to cause bursting of one of the balls. Dr. Rider enucleated both globes. This operation resulted in perfect restoration to health, an increase of thirty or forty pounds in weight, and complete relief from all symptoms of the disease, which were seriously threatening her life. A permanent enlargement of one gland remains, which causes no inconvenience. It is hardly possible there could have been real structural lesion here. Two cases of the above were significant in the fact that both, after suffering long, removed from the county, and began almost immediately to improve, finally recovering without medical aid. One, a very marked case, related to me by Dr. T. R. Bamber, of Carlton, was under treatment of various kinds at least six years. Two or three years after moving away, she was well. She declares it to be due to "change of location." The other, a young lady seen by Dr. John Taylor, of Holley, was gradually growing worse; but, after quitting the county, in a short time apparently recovered, and is now married. She was under no treatment here. There are no statistics to indicate that climate influences this disease, or tends to produce it, nor is the climate of this county known to vary materially from that in many others of the state; but the per cent. of carbonate of lime in the wells and springs throughout this section is unusually large; in some, extraordinary. The average in fourteen analyses, recently made in various parts of Albion, was twenty per cent. We may have no reason, however, to attribute any influence to such causes.

As regards treatment, the therapeutics of Graves' disease have, until recently, been more or less uncertain; due, principally, to the imperfect understanding as to its pathology. We have to deal with irritation of the sympathetic (accepting that theory,) dilatation of the blood-vessels, and symptoms resulting therefrom, a general neurotic condition, and the special indications of each individual case.

Resulting from experience and experiment, galvanization of the sympathetic and its cervical ganglia must rank first and most successful—not, however, overlooking any accompanying symptoms requiring relief. Upon the same theory, and from experience and observation, digitalis, iron, ergot and aconite are almost equally important. Some prefer strychnine with other remedies—one, lately, hydroiodic acid; and one case was reported as cured on milk. If there be a scrofulous diathesis, iodide of iron is excellent; otherwise, the dialyzed cannot be improved. Often the bromides form an important factor, and extirpation of the glands has, in a few extreme cases, been performed. There should be a nutritious diet maintained, and an avoidance of excitement and over-exertion, with plenty of rest and pure air and water. The main factor, however, contributing to successful treatment, as Dr. Rockwell says, is *persistence*,—persistence on the part of physician and patient. Understanding the usually accepted explanation as to its causes and the general basis of treatment, we must study the individual peculiarities, and select those remedies most applicable and best agreeing; and then, for a time, adhere with bull-dog tenacity, not looking for amelioration too soon.

So diversified are the cardinal symptoms in different cases, so varied their appearance and progress, the writer has collected the histories of as many as could be found, and deduced an average result in the main particulars. The number collected, many of which are incomplete, amounts to *two hundred and thirty-one*. It is from these reports that the writer has largely drawn in the preceding pages.

In this number, there were 197 females and thirty-four males, a proportion of six to one. This agrees with Gräfe. Flint places it higher, Ziemssen lower. Age was mentioned fifty-five times, the average being 27.7 years; youngest was two and one-half; oldest, sixty-six. Average of males was thirty-two and one-half years; youngest, fourteen; oldest, fifty-three.

Forty-seven reports made special reference to nervous complications. In thirty-one, or two-thirds, they were most prominent.

The *usual* pulse was mentioned thirty-two times. Seventy-four was the lowest given; 180, the highest; the average was 119. This does not include extremes, some of which could not be counted.

The time between the appearance of the first and second symptom of the triad, in fifteen cases given, averaged nearly two years, and varied from a few hours to thirty-one years.

As to the glandular swellings, one or both, nineteen specify. In ten, the right only was involved; in five, both; in four, the left.

In the 231 cases, dilatation of the pupil was mentioned four times. Hammond saw "a few." Russell records three in twenty-three.

In seventy-two, the first symptom noticeable was designated. In sixty-three, it was palpitation, or about six-sevenths. In eight, the goitre, or about one-eighth; and in only one, reported by Bartholow, did the proptosis first appear.

Of their absence, palpitation was wanting in only five. In eighty-one cases, ten had no goitre. In fifty-six cases, proptosis was absent ten times.

I can find but five fatal cases reported, not including sixteen post-mortems, where the history of the disease was not given. Of the five, one was by Trousseau, one by Greenamyer, one by Constantine Paul, one by Hammond, and one by Thomas; so it is safe to assume that a goodly majority, at least, of the 231 recovered partially or completely. Twenty are specified as having but partially recovered, and fifty-three completely. In nearly all of those improved, with the glands involved, more or less permanent enlargement remained. In only four did a serious heart complication follow; and as to the results of the exophthalmia, unfortunately, none are mentioned among the recoveries, save in a general way. How many resulted in partial or complete loss of sight, it would be interesting to know.

